# Rhegmatogenous Retinal Detachment in Ocular Toxoplasmosis: A Case Series

**DOI:** 10.7759/cureus.86714

**Published:** 2025-06-25

**Authors:** Sonny Caplash, Manuel Paez Escamilla, Sidra Zafar, Andrew W Eller, Jose Alain Sahel, Zachary Nadler, Aditya Uppuluri, Tatyana Beketova, Jay Chhablani, Fernando Arevalo, Marie Helene Errera

**Affiliations:** 1 Department of Ophthalmology, University of Pittsburgh, Pittsburgh, USA; 2 Department of Ophthalmology, Wilmer Eye Institute, Johns Hopkins Bayview Medical Center, Baltimore, USA

**Keywords:** pars plana vitrectomy, retinal detachment, retinal tear, scleral buckle, toxoplasmosis

## Abstract

*Toxoplasma gondii* causes ocular toxoplasmosis (OT) with potential retinal complications like rhegmatogenous retinal detachment (RRD), and retinal breaks (RB). While various management strategies exist, there is no universally accepted standard of care, and the most common combination procedure is scleral buckle (SB), pars plana vitrectomy (PPV), and silicone oil. The purpose of this case series is to evaluate and discuss the risk factors, phenotypes, management, and outcomes of rhegmatogenous RRD in six eyes (five patients) with OT from retrospective chart review. The age range was broad (27-77 years old), with four patients over the age of 50. RRDs were caused by one retinal tear per eye in two out of five eyes (40%), and ≥2 tears in three out of five eyes (60%). No retinal tears were detected in one eye. We reported 77% of superior retinal tears, and the incidence of macula-off RRD was 66% (four out of six eyes). Three eyes underwent PPV with gas tamponade, and two eyes underwent SB with PPV and gas. One patient declined surgery. The five eyes affected with RRD that underwent surgery had a retinal re-attachment after one surgery (100%) with a mean follow-up of 26 months. Four out of five eyes had visual improvement, and one eye lost one line of vision (20/200 to 20/400). An age older than 50 years and CT or inactive acquired OT appear to be risk factors for RRD. Depending on the location of retinal tears, particularly inferior, or the chronicity of RRD, an SB can be useful to add to PPV.

## Introduction

Toxoplasmosis is a parasitic infection caused by the protozoan *Toxoplasma gondii*. *T. gondii* is a coccidian parasite whose definitive hosts are domestic cats and their relatives. The parasite’s eventual oocyst is fecally excreted into the environment and encounters a wide diversity of intermediate hosts. Humans become infected by either direct contact with these fecal oocytes or through the consumption of another intermediate host with a localized tissue cyst. Ingestion of undercooked meat, contaminated water, and transplacental spread represent two of the most common forms of transmission for *T. gondii,* leading to acquired and congenital toxoplasmosis, respectively. Once infected, humans can harbor a *T. gondii* tissue cyst for years with acute infection and inflammation precipitated by relative immunosuppression [1].

Congenital toxoplasmosis (CT) has an estimated incidence of 190,000 cases annually, consequently accounting for 1.2 million disability-adjusted life years annually [1]. Data from a systematic review estimates a global IgG seroprevalence of *T. gondii* of roughly one-third [2]. It is important to note that accurate estimation of CT worldwide is limited by the overall scarcity of comprehensive prenatal screening programs. This is exemplified in data from the European Union in 2020, where the notification rate for human congenital toxoplasmosis across Europe was 5.1 per 100,000 live births, where France accounted for 82.7% of all reported cases because of compulsory screening of pregnant women [3,4].

The seroprevalence of toxoplasmosis is variable between different regions of the world. Data indicate that the highest seroprevalence is found in the Americas, Eastern Mediterranean, and Africa. More specifically, Ethiopia, Gabon, and Brazil were found to have the highest seroprevalence among countries with eligible data, with rates of 64.2%, 56.7%, and 53.8%, respectively [4]. In contrast, the United States has an estimated seroprevalence that ranges from 10% to 20% [4,5].

Ocular toxoplasmosis (OT) presents classically with an active retinitis adjacent to a retinochoroidal scar and is often associated with vitritis, macular edema, and epiretinal membrane. There is also often a nodular retinitis [5,6]. A rare but important downstream sequela of OT is rhegmatogenous retinal detachment (RRD), which can lead to significant ocular morbidity.

In the general population, the annual international incidence of RRD is estimated to be 12.17 per 100,000 [5]. In patients with OT, the incidence of RRD has been cited to be anywhere from 0.6% to 11.4% [6-10]. A recent meta-analysis showed that within one year of experiencing retinochoroiditis, the risk of developing RD or breaks is approximately 62% of the patients [10].

Although the mechanism of RRD in OT is not fully understood, it is thought that vitritis leads to vitreous liquefaction and traction, which results in RRD and/or tractional retinal detachment (TRD) [11]. Given the relatively low prevalence of OT in the United States relative to other parts of the world, there is an overall lower probability of encountering RRD associated with OT.

The purpose of this report was to evaluate and discuss the risk factors, phenotypes, management, and outcomes of RRD in patients with a history of OT, which is a rare complication. The present series was further prompted by an editorial by Cunningham et al., reminding us that in the setting of uveitis, RRD occurs most often in eyes with infectious retinitis [12].

## Case presentation

This was a retrospective, non-comparative review of the charts of OT cases at two academic centers, where patients presented either to the retina or the uveitis clinics. Diagnosis of OT was primarily clinical, based on the presence of characteristic retinochoroidal scars, adjacent retinitis, and/or vitritis [13]. Three patients underwent confirmatory polymerase chain reaction (PCR) testing. This retrospective series was conducted in accordance with the tenets of the Declaration of Helsinki under a protocol that was approved by the University of Pittsburgh Medical Center Institutional Review Board (STUDYMOD19010072-006).

Case 1

A 25-year-old female immigrant from South America presented to our clinic for decreased vision bilaterally. Her symptoms started four years earlier with her right eye when she initially noticed photopsias, followed by painless, subacute vision loss over one week. Since that time, her vision in the right eye has been poor, but nonprogressive. Two years later, when the patient was pregnant, at four months of gestation, she woke up with acute decreased vision in the left eye. The patient denied any previous ocular surgery.

No medical records were available regarding the pregnancy abroad, and the patient was not clear on details other than the baby was not "premature". Therefore, unclear whether a screening of TORCH (toxoplasmosis, rubella, cytomegalovirus, herpes simplex, and HIV) was done at the time of pregnancy. The toxoplasmosis serology was performed one week prior to the retinal detachment surgery and showed positive *Toxoplasma* IgG AB at 140.00 IU/mL (high) and negative T*oxoplasma* AB (IgM) <0.8 IU/mL. The rest of the uveitis work-up showed HIV-1 antigen and HIV-1/HIV-2 antibodies were not detected, angiotensin-converting enzyme (ACE) was normal, QuantiFERON(R) TB Gold was negative, and no acute pulmonary abnormality was seen on the Chest X-ray. Her vision remained poor since that incident, but non-progressive. She did not seek medical care for either of these episodes.

**Figure 1:**
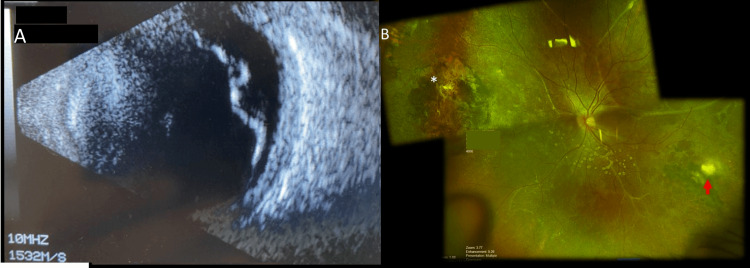
Ultrasound and fundus images (Case 1) (A) B scan ultrasound showing total retinal detachment in left eye; (B) Wide fields fundus picture image showing retina attached, widespread subretinal gliosis with superonasal laser scar (asterisk) and inferotemporal toxoplasmosis retinochoroidal scar (red arrow).

On examination, the patient’s best corrected visual acuity (BCVA) was 20/500 in the right eye and light perception in the left eye. There was a relative afferent pupillary defect in the left eye. Intraocular pressure was 12 mmHg in each eye, and extraocular movements were intact. Slit lamp examination showed a normal anterior segment exam in the right eye. The left eye demonstrated a pupillary membrane overlying the pupil. Dilated fundus examination of the right eye demonstrated temporal disc pallor with a large macular retinochoroidal scar. B-scan ultrasonography of the left eye revealed a retinal detachment (RD) (Figure 1A).

The patient subsequently consented to left eye phacoemulsification and pars plana vitrectomy (PPV). Intraoperative evaluation of the left eye fundus demonstrated a posterior vitreous detachment present, a total RD, a horseshoe tear (HST) superotemporally, and an inferotemporal retinochoroidal scar (Figure 1B). She underwent 25-gauge PPV with endolaser, perfluorocarbon, and 20% SF6 gas. Postoperatively, the patient’s final BCVA was 20/200 in the right eye, and she could count fingers (CF) at 2 feet in the left eye at last follow-up at 13 months.

Case 2

A 52-year-old male patient was referred by an outside provider with an acute onset of decreased vision in the left eye. The patient reportedly had a history of bilateral CT as well as cataract surgery in the left eye several years prior. On presentation, the patient endorsed a two-day history of flashes and floaters in the left eye with subsequent curtain of darkness. The patient denied any other past medical history.

On exam, the patient’s visual acuity at distance in the right eye was count fingers at 1 foot. In the left eye, it was 20/100. Pupils were pharmacologically dilated. Intraocular pressure was normal in both eyes. Confrontational visual fields revealed superior and temporal constriction. The anterior segment in the right eye was remarkable for 3+ nuclear sclerosis. The anterior segment in the left eye was largely unremarkable, with a well-centered posterior capsular intraocular lens. The right eye vitreous was clear. The left eye vitreous demonstrated heme and pigment. Dilated fundus examination of the right eye showed macular retinochoroidal scars. Dilated fundus examination of the left eye showed a macula-off RD with a tear seen at 9 o’clock.

**Figure 2:**
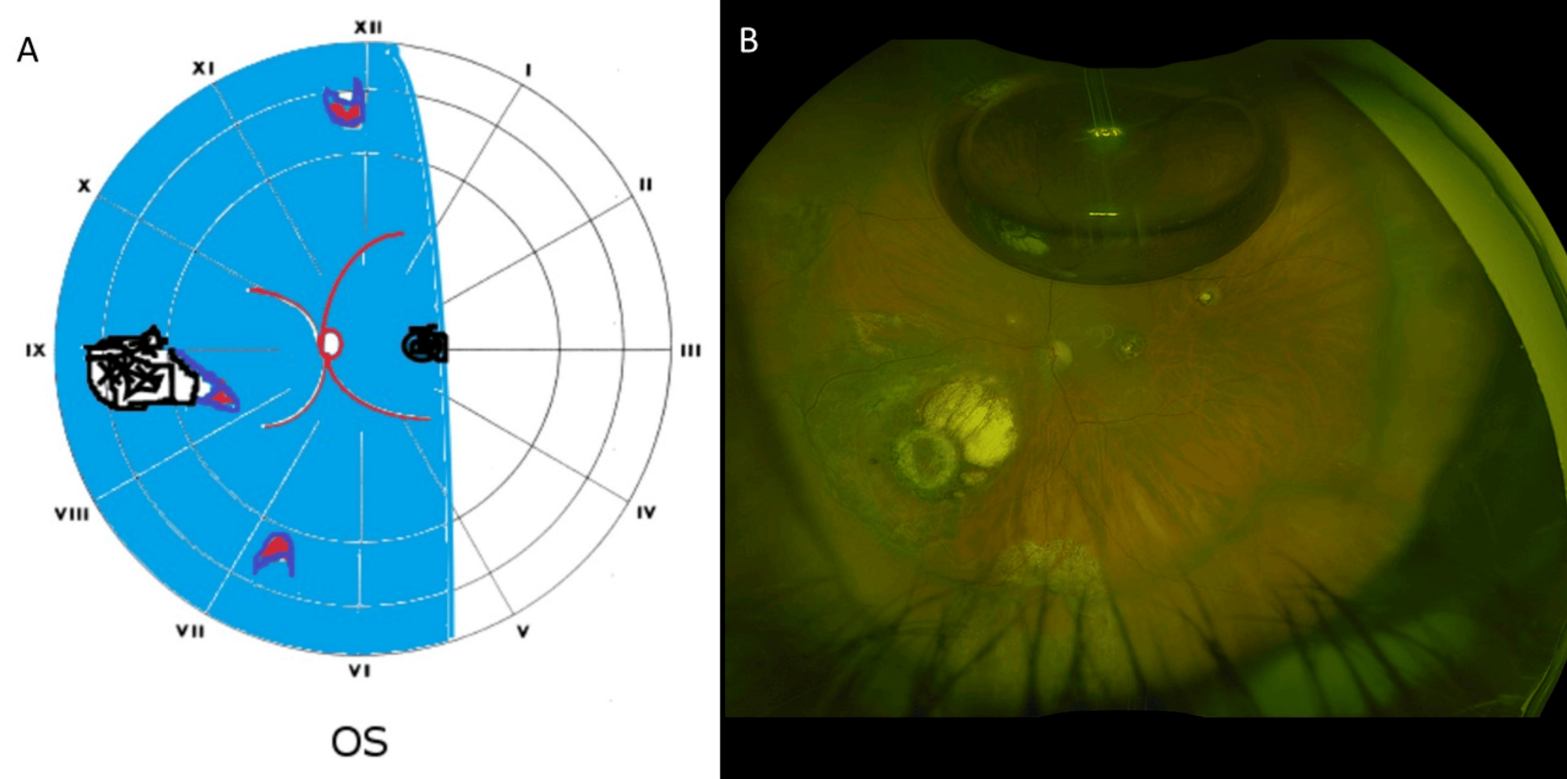
Preoperative drawing and postoperative fundus image of Case 2 (A) Preoperative drawing showing the nasal rhegmatogenous retinal detachment bisecting the macula with toxoplasmosis retinochoroidal scars at macula and at 9 o’clock, and x3 retinal breaks at 6, 12, and at the border of the toxoplasmosis retinochoroidal scar at 9 o’clock. (B) Wide-field fundus image showing retina attached behind 10% gas fill, with toxoplasmosis retinochoroidal scars at macula. Note the laser pigmented marks at 9 o'clock surrounding the retinochoroidal scar and around the retinal breaks at 6 and 9 o'clock.

Intraoperatively, the patient was found to have a nasal RRD with HSTs at 8, 9 at the anterior edge to an old retinochoroidal scar, and 12 o’clock. An SB was placed, followed by PPV with endolaser. The choice of SB was made because of an inferior retinal tear. The eye was filled with 14% C3F8. The postoperative course was uncomplicated, and the patient’s final BCVA in the left eye was 20/60. Figure 2 shows the postoperative fundus image with a retina attached behind the 10% gas fill and the inferonasal retinochoroidal scars surrounded by laser and macular, and temporal old pigmented toxoplasmic retinochoroidal pigmented scars.

Case 3

A 54-year-old male patient with a history of CT presented to the clinic with decreased vision in the right eye. The patient was known to the clinic and had previously documented retinochoroidal scars; more specifically, one peripheral retinochoroidal scar in the right eye and a macular retinochoroidal scar in the left eye. The patient had a history of previous flares of toxoplasmosis with vitritis and retinitis adjacent to retinochoroidal scarring; these were previously treated with oral trimethoprim-sulfamethoxazole.

On the day of presentation, the patient reported blurry vision in the right eye. The patient’s vision was 20/25 in the right eye and 20/200 in the left eye. The patient’s intraocular pressures were normal. Pupillary exam was normal and without a relative afferent pupillary defect. There were no abnormalities on the motility exam and confrontational visual fields. The patient’s slit lamp examination in the right eye revealed fine inferior keratic precipitates on the corneal endothelium and 2+ cell and flare in the anterior chamber. The patient’s left eye was within normal limits. On dilated fundus exam, the right eye exhibited 1-2+ vitreous cells, retinal vessel sheathing inferiorly, and an inferior retinochoroidal scar with an adjacent retinal infiltrate just inferior to the scar. Dilated fundus exam of the left eye revealed a macular retinochoroidal scar.

**Figure 3:**
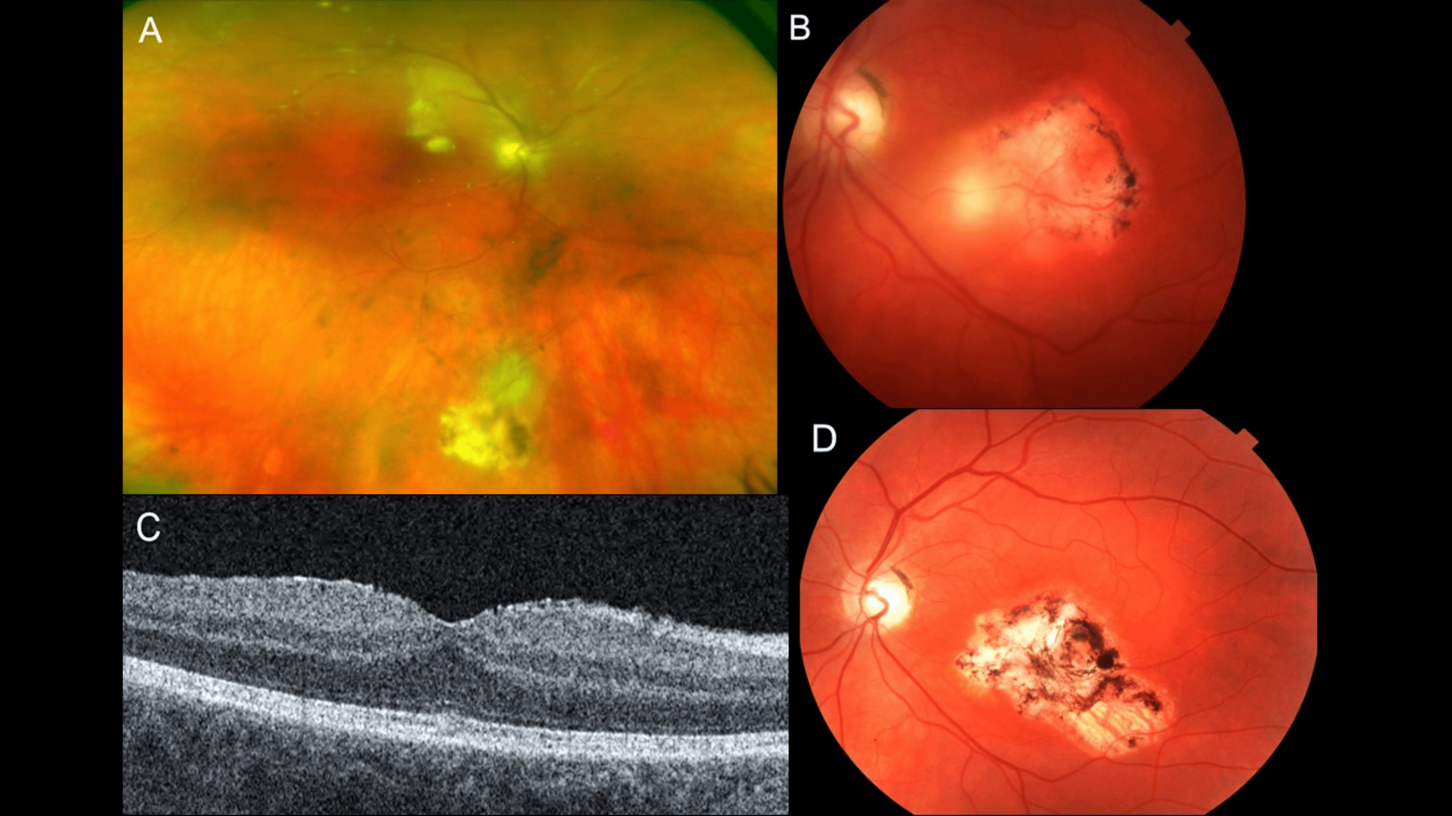
Imaging findings of Case 3 (A) Wide-field fundus photo of the right eye shows a hazy view of the fundus with a chorioretinal scar in the inferior mid-periphery with active recurrence of toxoplasmosis on its superior border. (B) Fundus photo of the left eye shows slightly hazy media with a large, central chorioretinal scar. There is a white chorioretinal infiltrate on the nasal aspect of the lesion consistent with recurrence of active toxoplasmosis. (C) Optical coherence tomography of the right eye showing intact macula. (D) Fundus photo of the left eye showing a healed chorioretinal scar.

The patient was prescribed oral trimethoprim, sulfamethoxazole double-strength (DS), twice daily for six weeks, and prednisolone acetate four times daily in the right eye. On follow-up examination, the patient continued to have vitritis and persistence of retinal infiltrate in the right eye. Intravitreal clindamycin (1 mg/0.1 mL) was then given in the right eye. Two days later, the patient presented with decreased vision and was found to have a vitreous hemorrhage with an associated RRD in the right eye (Figure 3A). The patient underwent PPV with endolaser, air-fluid exchange, and SF6 gas injection. Intraoperatively, the patient was found to have a 2 o’clock tear from 8:30 to 10:30 with associated subretinal fluid, and one tear at 12 o’clock close to the ora serrata. Vitreous sampling was performed at the time of surgery and was PCR positive for *T. gondii*. Postoperatively, the patient’s final visual acuity in the affected eye was 20/15 with an intact foveal contour on optical coherence tomography (OCT) (Figure 3C). The patient had one subsequent reactivation in the left eye (Figure 3B) one year later that was successfully treated with trimethoprim-sulfamethoxazole and topical prednisolone acetate (Figure 3D).

One year after this, and two years after the diagnosis of RD in the right eye, the patient developed a RRD in the left eye. Of note, there was no preceding intravitreal injection. Examination revealed a BCVA of 20/25 in the right eye and 20/200 in the left eye. Fundus examination of the right eye revealed a previous retinochoroidal scar inferiorly at 6 o’clock without any active borders. The previous HSTs from 8:00 to 10:30 and a round hole at 12 o’clock were seen with surrounding endolaser, and the retina appeared flat. Dilated fundus examination of the left eye revealed 1-2+ vitritis, the patient’s previously documented macular retinochoroidal scar with retinitis at the inferior border. There was also a temporal retinochoroidal scar without any associated activity. The patient subsequently underwent a PPV with endolaser photocoagulation and 20% SF6 gas. Intraoperative examination revealed retinal breaks at 11:00, 12:00, 1:00, and 1:30 o’clock. Notably, the breaks at 1:00 and 1:30 o’clock were associated with subretinal fluid and the patient’s temporal RRD. The patient’s final BCVA was 20/100 in the left eye.

Case 4

A 77-year-old male patient with a history of graft versus host disease presented with two months of progressively worsening floaters and blurred vision in the left eye. At presentation, his BCVA was 20/400 in the right eye and reduced to CF in the left eye. Examination of the right eye was unremarkable. The left eye slit lamp examination was remarkable for diffuse vitreous veils and 2+ anterior vitreous cells in the left eye. Dilated fundus examination of the left eye revealed 2+ vitreous haze, a thickened, hypopigmented lesion in the macula, and a retinochoroidal scar along the inferior arcade. The patient underwent a diagnostic anterior chamber paracentesis for viral (herpes simplex virus (HSV), varicella zoster virus (VZV), and cytomegalovirus (CMV)) and *Toxoplasma* PCR testing.

Intravitreal foscarnet was administered, and he was also started on oral trimethoprim-sulfamethoxazole DS twice daily and valaciclovir 2 g three times daily. Approximately 48 hours from presentation, his aqueous sample returned negative for viral etiologies. He also tested negative for syphilis and toxocariasis and positive for toxoplasmosis IgM and IgG serology. At this point, the valaciclovir was discontinued, and the patient continued on oral trimethoprim-sulfamethoxazole DS. Oral prednisone 40 mg was also started. A complete metabolic panel was done at the initial presentation, and liver function test (LFT) was normal.

**Figure 4:**
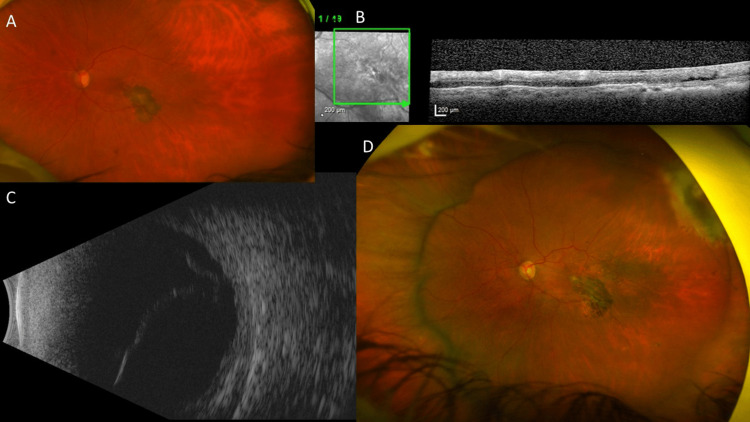
Imaging findings of Case 4 (A) Color fundus photograph showing the toxoplasmosis retinochoroidal scar at the macula. (B) Optical coherence tomography showing the toxoplasmosis retinochoroidal scar with mild subretinal fluid. (C) B scan ultrasound showing the retinal detachment in left eye. (D) Color fundus photograph showing the toxoplasmosis retinochoroidal scar at the macula and the buckling effect nasally.

Over the next 14 months of follow-up, the patient’s BCVA stayed stable at around 20/400. Figures 4A, 4B show the toxoplasmosis retinochoroidal scar at the macula with mild subretinal fluid. His clinical course was complicated by the development of macular edema that required a posterior sub-tenon’s triamcinolone injection (Figure 4B). Unfortunately, due to COVID-19-related restrictions, the patient was lost to follow-up until 24 months later. He had noted a significant decline in his vision in his left eye one to two months prior to re-presentation. His left eye examination was notable for 360 posterior synechiae, a quiet AC, and a dense, brunescent cataract. The intraocular pressure was normal in both eyes, 12 mmHg in the right eye and 7 mmHg in the left eye. The B-scan ultrasound showed a total RD (Figure 4C). After an uncomplicated cataract surgery with intraocular lens implant, the vision was reduced to light perception in the left eye, and his exam was notable for a total bullous macula-involving RD and a small retinal break, temporally. The patient subsequently underwent an SB with 23-gauge PPV, endodiathermy, and C3F8 gas for RD repair. At this last follow-up, approximately 18 months from RD repair surgery, his retina remained attached (Figure 4D). BCVA was CF in the left eye, and he was on prophylactic treatment, oral trimethoprim-sulfamethoxazole prophylaxis every other day.

Case 5

**Figure 5:**
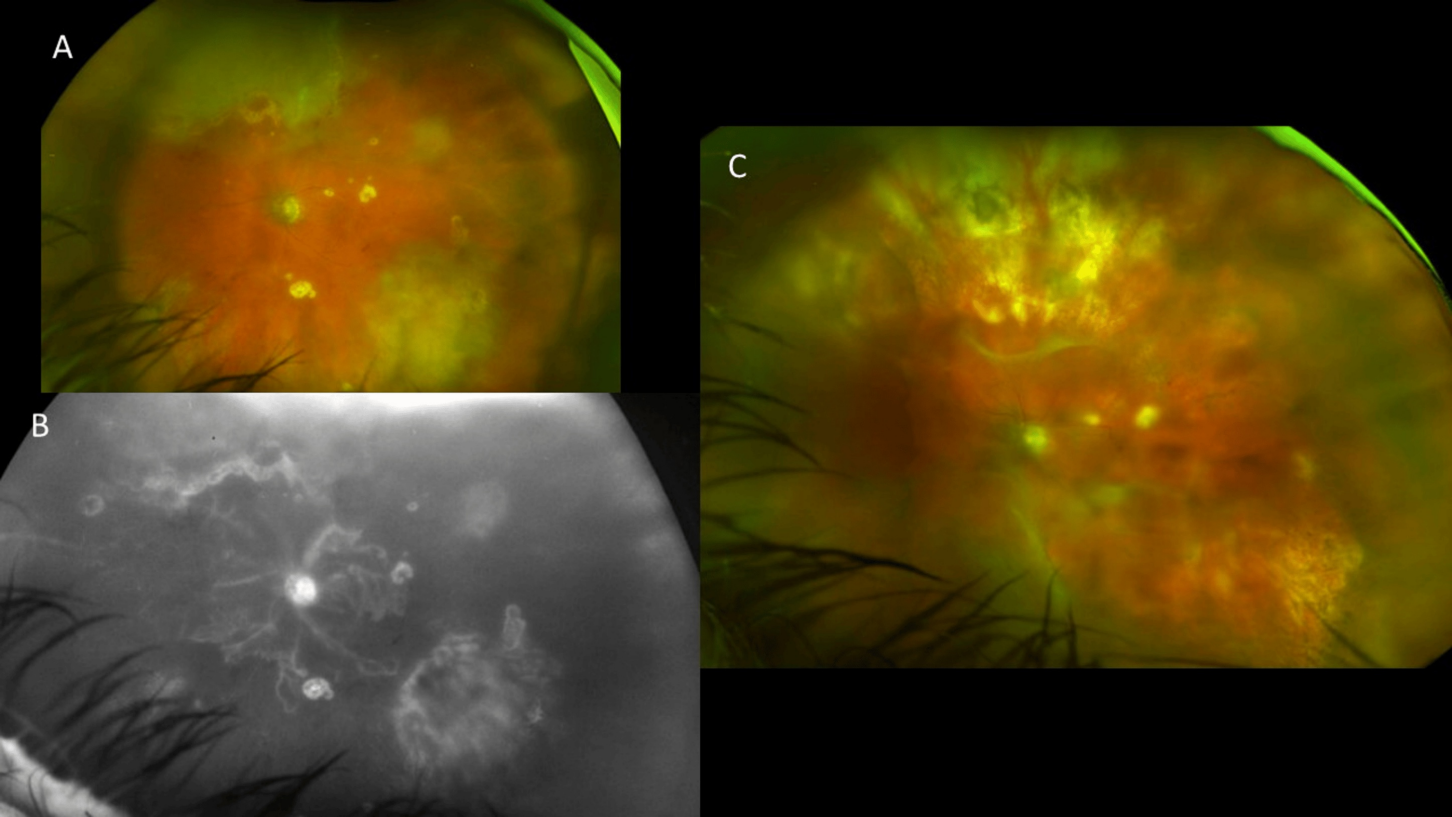
Imaging findings for Case 5. (A) Color fundus photograph showing an extensive inferotemporal and superonasal retinitis or retinochoroiditis with punched out smaller chorioretinitis lesions at the posterior pole. (B) Fundus fluorescein angiography revealing an extensive occlusive retinal vasculitis and hyperfluorescent retinochoroiditis lesions in the left eye. (C) During follow-up at eight months, color fundus photograph showing a subtotal retinal detachment, macula-off in the same left eye.

A 71-year-old male patient without relevant past medical history presented with a loss of vision in the left eye (BCVA 20/150). Examination of the left eye showed a hypertensive granulomatous panuveitis with extensive inferotemporal, superonasal retinochoroiditis, and smaller punctuate macular lesions (Figure 5A). The fundus fluorescein angiography revealed an extensive occlusive retinal vasculitis and hyperfluorescent retinochoroiditis in the left eye (Figure 5B). A diagnostic PPV was negative for CMV, HSV, VZV by PCR and negative for ocular lymphoma (no *MYD88 L265P* mutation detected, negative by flow cytometric immunophenotypic studies), IL-6>IL-10.

The patient was diagnosed with ocular toxoplasmosis because of IgG (+) (>400.00 high) and highly positive quantitative PCR (qPCR) of the eye aqueous humor (48,400 copies/mL). He was treated on prolonged sulfamethoxazole-trimethoprim (Bactrim DS) 800-160 mg two times a day for nine weeks, as well as isoniazide 300 mg + rifampicin 600 mg daily for three months due to a positive QuantiFERON-TB Gold test with healing of the retinochoroiditis. During follow-up at eight months, he was diagnosed with subtotal RD, macula-off in the left eye (Figure 5C). The fundus view was hazy due to pigment on the intraocular lens, and no retinal tear was detected. The patient declined retinal repair by surgery due to irreversible vision loss prior to the RRD. 

Consolidated results

Demographics

Our case series demonstrates five cases (six eyes affected with RRD and OT), four males and one female. The age range was broad (27-77 years old), with three patients over the age of 50. Two patients had CT, one had an acquired OT, and two had a recent active OT. Two out of five patients were myopes. The mean follow-up was 26 months (range 3-48 months). Data have been given in Table 1.

**Table 1:** Demographical and clinical data of the cases CF: counting fingers; CT: congenital toxoplasmosis; DD: disc diameter HM: hand motion, HST: horse shoe tear; IT : inferotemporal ; LP: light perception; M: male; F: female; PPV: pars plana vitrectomy; RRD: rhegmatogenous retinal detachment; SB: scleral buckle; ST: superotemporal; VA: visual acuity (Snellen); OS: oculus sinister (left eye); OD: ocular dexter (right eye)

Case number	Age (years)/sex	Eye	Diagnosis	Type of toxoplasmosis lesion	Location of toxoplasmosis lesion; zone 1 (macula); zone 2-3 (outside vascular arcades)	Preoperative VA	Surgery	Final anatomical result	Follow-up (month)	Final VA
Case 1	25/ F	OS	RRD macula off x1 HST (ST)	CT	IT (<1DD) zone 1	LP	25G PPV, endolaser, perfluorocarbon, 20% SF6	Attached retina	13	CF
Case 2	52/ M	OS	RRD macula off x3 HST (8, 9 at edge of retinochoroidal scar, 12 o’ clock)	CT	9 o’clock + macula (<1DD) zone 1	CF	25G PPV, endolaser, SB, 14% C3F8	Attached retina	3	20/60
Case 3	54/ M	OD	RRD macula on x2 tears (2 o’clock from 8:30 to 10:30- 1 tear at 12 o’clock)	Acquired acute toxoplasmosis, vitritis	Inferior (1DD active lesion) zone 2	20/100	PPV, endolaser, SF6 gas	Attached retina	48	20/20
		OS	RRD macula on x4 breaks at 11:00, 12:00, 1:00, 1:30 o’clock	Acquired acute toxoplasmosis, vitritis	Temporal (1DD active lesion) zone 1	20/200	PPV, endolaser, 20% SF6	Attached retina	48	20/400
Case 4	77/ M	OS	Chronic RRD macula on, x1 break, temporal	Recent acquired toxoplasmosis	inferior vascular arcade (2DD), zone 1	CF	SB with 23-gauge PPV, endodiathermy and C3F8 gas	Attached retina	18	CF
Case 5	71/M	OS	Subtotal RRD macula off, no retinal tear seen (hazy view through IOL)	Recent acquired toxoplasmosis	Extensive retinochoroitisi <20DD superonasal and inferotemporal (zones 2-3), punctate lesions zone 1	20/150	No surgery for RRD repair (PPV at diagnostic, 8 months prior to RRD)	Detached retina	8	HM

Clinical findings/Location of Toxoplasmic Retinochoroiditis Scar

All presented cases had RRDs, and only one occurred in the setting of active ocular toxoplasmosis. In our first case, there was already a posterior vitreous detachment, and one case had a prior diagnostic vitrectomy. Among the five patients of this case series, there were six RRDs (six eyes affected). The location of the retinal tears and retinochoroiditis lesions is summarized in Table 1. In one eye, no retinal tear was detected through the hazy intraocular lens. RDs were caused by one retina tear per eye in two out of five eyes affected (40%), and ≥2 tears per eye in three out of five eyes (60%). In only one eye (1/ 5 eyes, 20%), one tear was found adjacent to one retinochoroidal toxoplasmosis scar. In two eyes affected with RRD (2/5 eyes, 40%), retinal tears occurred in areas of previous retinochoroiditis, but not exclusively limited to these areas. Among a total of 13 retinal tears in five eyes, 10 tears were located superiorly (77%). No eyes had proliferative vitreoretinopathy (PVR) reported, although in Case 4, a chronic RRD was reported. Of the five patients affected with six RRDs, four of the six eyes (66%) initially had macular involvement.

Surgical Treatments

With regards to surgical management, three cases underwent PPV with gas tamponade, and two cases underwent SB with PPV and gas. The choice of SB was made due to inferior retinal break (Case 2) or chronic RRD (Case 4).

Outcomes

Among the five eyes affected with RRD that underwent a RD repair, all had a retinal re-attachment after one surgery (100%), and the retinochoroidal inflammation subsided after surgery and antibiotic treatment. In terms of visual outcomes, when comparing baseline to final BCVA, four out of five eyes that had an RRD repair had visual improvement at final follow-up, and one eye lost one line of vision (20/200 to 20/400) (left eye, Case 3).

## Discussion

Risk factors for RRD in OT

Overall, the outcomes in this series suggest that OT can lead to RRD. One large retrospective study estimated that roughly 8% of all uveitis associated with RRD was due to OT [9]. While the overall incidence of RRD among OT is relatively low, *T. gondii’*s high seroprevalence globally makes it a substantial source of ocular morbidity.

Data on risk factors that predispose eyes with OT to the occurrence of RRD is limited, as its overall incidence is relatively low. Data from several studies implicate myopia, active inflammation, significant vitritis, female sex, and younger patients [9-11]. In our study, only 20% of the patients affected with RRD and OT were female (one case), and 80% of patients were 52 years of age or older (four out of five patients). Finally, only one RRD (20%) occurred in the setting of active OT.

Retinal tears, number, and location

Review of current literature reveals a pattern of primarily superior retinal breaks [8]. Our current case series is in accordance with this since we report 77% of superior retina tears. With regards to macular involvement of RRD, one prospective study implicates an incidence of 50% among OT patients who present with RRD [5]. Our current study reports the incidence of macula off RRD to be 66% (four out of six eyes).

With regards to the location of the retinal tear in relation to previous areas of inflammation, the data is limited. Two retrospective case series do shed some light on the association of retinal tears in OT [8,9]. Faridi et al. examined 35 eyes of 28 patients [11]. Four of their patients developed an RD. Notably, two out of four patients had a documented tear associated with retinochoroiditis. One patient had a superior RD, one had an inferior RD, one had a total RD, and lastly, one patient had an initial macula-on detachment, without location specified. Two of the four patients had courses complicated by tractional RD (TRD) [11]. Bosch-Driessen et al. performed a retrospective chart review of 150 OT patients; 11 out of 16 patients with RD in the setting of OT had documented retinal breaks [14]. Among these 11 patients, six had a tear in the superior retina. Five had a retinal tear adjacent to a toxoplasmic lesion. In our cases, only one retinal tear was found at the anterior edge of a retinochoroidal toxoplasmosis scar (20%).

The current understanding of the mechanism of RRD in OT is that vitreous inflammation can lead to vitreous contraction, leaving the vitreous partially attached to the retina, and causing tractional forces [4-6]. However, only one of our six eyes affected with RRD was found with active OT. Therefore, the mechanism of vitritis cannot explain most of our cases. Two of our five patients (2/6 eyes) received an intravitreal injection, which is a risk factor for RRD, especially in an inflamed eye, and one eye had a previous diagnostic PPV at the onset of OT. The same eye had an extensive area of retinochoroiditis >20 DD.

Previous studies noted the relatively young age of patients who present with RRD with OT and seemed to support a tractional hypothesis given their relatively formed and adherent vitreous [4-6]. In our case series, that hypothesis is not relevant, since most of the patients with RRDs and OT were 52 years of age or older. It is important to note that while tears and breaks in OT can occur in areas of previous retinochoroiditis, they are not exclusively limited to these areas [4-6]. When comparing with our case series, in two eyes affected with RRD (40%), tears in OT occurred in areas of previous retinochoroiditis, but even in those two eyes, other retinal breaks were noted, not exclusively limited to these areas.

Surgical approach for RRD treatment

Data from Moreira et al. illuminates the efficacy of various surgical approaches [10]. A total of 31 surgeries were performed by them, and the retina was reattached in 15 patients (68.2%) immediately after the first surgery and in 20 patients (90.9%) after subsequent surgeries.

Five different isolated or combined procedures were performed, and the most common combination procedure was SB, PPV, and silicone oil (n=10 patients; 45.4%). The technique that achieved the highest rate of primary retinal reattachment was PPV and silicone oil (4 patients; 100.0%), and all study participants who underwent SB alone (n=4) or PPV with gas infusion (n=1) exhibited functional improvement [10].

Faridi et al. also evaluated the surgical management of patients with OT-associated RD [11]. Of the four patients with RD in their study, three underwent initial PPV and one underwent laser retinopexy. The patient who underwent laser retinopexy did so in the setting of a single break with minimal vitritis. Two of the other three patients were complicated by recurrent TRDs requiring eventual SB or long-term tamponade with silicone oil. All four patients have successful, eventual anatomic outcomes. The three surgical patients had poor visual outcomes ranging from 20/400 to CF at 1’. The one patient who underwent laser retinopexy had an excellent visual outcome at 20/15 [11]. Bosch-Driessen et al. looked at the surgical approach for RD in OT [14]. In their study, nine patients had RD, of whom four underwent SB alone, one underwent PPV, one underwent PPV/SO, and one underwent cryopexy. Surgical management was withheld in the remaining two patients. In our case series, three cases underwent PPV with gas tamponade, and two cases underwent SB with PPV and gas. The five eyes affected with RRD who underwent a RD repair had a retinal re-attachment after one surgery (100%), which is in accordance with most studies that demonstrate high rates of eventual re-attachment [10,15-20].

Final VA varied. Expectedly, in patients where surgical management was deferred, final VA was poor at either no light perception (NLP) or light perception (LP). In patients who underwent intervention, final VA varied from 20/20 to CF. Final BCVA improved in four out of five eyes after RD repair, with the one not improving being due to the development of a macular scar. Amaral et al. in their meta-analysis study (14 studies, 86 patients with RD and/or break) showed that surgical treatment can substantially improve BCVA, with an average increase of 0.60 LogMAR [16]. Our final visual outcomes were similar to those published by others; the BCVA was 20/15 (one patient, right eye, Case 3), and the worst was CF in two patients (Case 1 and Case 4). Both cases 1 and 4 presented with macula-off RRDs and a delay in RRD repair. Case 4 presented with a retinochoroidal scar at the macula, and Case 1 with subretinal gliosis from chronic RRD.

Complications

Postoperatively, patients can suffer from ocular hypertension or, more rarely, hypotony [16]. All studies reviewed demonstrated that in some patients with OT, the post-surgical course is often complicated by epiretinal membrane formation, macular hole, and proliferative vitreoretinopathy (PVR) [16,17]. These two sequelae can lead to both RRDs and TRDs. Data on the incidence of further surgical intervention ranges from one-third to one-half of patients with OT and RD [16,17]. Data from Moreira et al. showed that roughly one-third of patients required further surgical intervention, suggesting that patients with SB alone were most likely to re-present with a recurrent RRD [10].

While surgical data can be hard to interpret and extrapolate given the inter-case heterogeneity, current published data suggest that complete removal of vitreous traction by PPV and gas or silicone oil tamponade ± SB are required to stabilize the retina of patients with OT.

## Conclusions

RDs are a rare but serious source of ocular morbidity in patients with OT. The vitreous inflammation caused by toxoplasmosis may lead to vitreous traction, causing RRD, specifically in patients with notable risk factors for RD, such as myopic RD. This case series of six eyes of five patients affected with OT and RRD showed that age older than 50 years and CT or inactive acquired OT are also risk factors. With regards to surgical management, three cases underwent PPV with gas tamponade, and two cases underwent SB with PPV and gas. All our patients treated with this surgical approach had success with retinal re-attachment of 100% after one surgery, with notable improvements in VA. Our case series shows that depending on the location of retinal tear, particularly inferior or the chronicity of RRD, an SB can be useful to add to PPV. We recognize the intrinsic limitations of this report given its small sample size. Further prospective trials are recommended to substantiate the presented data.
